# Aortic valve replacement in a bicuspid aortic valve patient followed by reoperation for ascending aorta rupture: a case report

**DOI:** 10.3389/fcvm.2024.1471686

**Published:** 2024-12-11

**Authors:** Yuehang Yang, Xinyi Liu, Junwei Liu, Jiawei Shi, Huadong Li

**Affiliations:** ^1^ Department of Cardiovascular Surgery, Union Hospital, Tongji Medical College, Huazhong University of Science and Technology, Wuhan, China; ^2^ Department of Cardiovascular Surgery, Beijing Anzhen Hospital, Capital Medical University, Beijing, China

**Keywords:** ascending aortic dilatation, aortic rupture, cardiac reoperation, bicuspid aortic valve, aortic valve replacement

## Abstract

Bicuspid aortic valve (BAV), the most common congenital cardiac anomaly, predisposes individuals to aortic stenosis and regurgitation due to valve degeneration. Abnormal hemodynamics, arterial wall characteristics, and genetic factors contribute to ascending aorta dilatation, potentially leading to severe complications like aortic dissection. Presently, the most recent guidelines propose that individuals with BAV requiring valve replacement due to valve dysfunction should undergo simultaneous replacement of the ascending aorta when the diameter of aortic dilatation exceeds 4.5 cm. A 60-year-old female patient previously underwent mechanical aortic valve replacement and ascending aortoplasty at our center due to aortic stenosis and a 4.3 cm diameter ascending aorta. In the sixth postoperative year, she was readmitted due to ascending aorta rupture, resulting in blood entering the right atrium and causing acute right heart failure. We performed repair of the rupture and concomitant ascending aorta replacement to prevent further exacerbation of the patient's condition.

## Introduction

The bicuspid aortic valve (BAV) represents a prevalent congenital malformation arising from the fusion of the two aortic valve cusps in the trileaflet aortic valve (TAV). Apart from the symptoms attributable to the valve, such as stenosis and valvular insufficiency, the presence of abnormal hemodynamics amplifies the risk of aortic dilatation by 86% (1). According to research statistics, although the overall incidence of aortic dissection in patients with BAV is relatively low, it remains significantly higher than in the general population (2). Consequently, in individuals who present with BAV along with ascending aortic dilatation, determining the optimal timing and surgical approach is highly important. Previous research findings indicate that following aortic valve replacement in BAV patients, the probability of aortic expansion diminishes, approaching that observed in TAV patients. Simultaneously, the prevailing perspective regarding aortic replacement in BAV patients currently tends toward a conservative approach. Aortic replacement is deemed reasonable only in situations where the BAV is coupled with valvular dysfunction requiring valve replacement and the diameter of aortic dilatation exceeds 4.5 cm (3). We report a rare case of rupture of the ascending aorta into the right atrium at 6 years after aortic valve replacement and ascending aortoplasty.

## Case description

A 66-year-old female patient was identified six years ago with adhesions at the junction of the left and right coronary valves of the trileaflet aortic valve, resulting in a bicuspid deformity and severe stenosis of the aortic valve. Simultaneously, there was concurrent aneurysmal dilatation of the ascending aorta. Upon admission, the patient did not manifest any discernible symptoms. During physical examination, her heart rate was recorded at 84 beats per/minute, and her blood pressure was measured at 143/101 mmHg. Transthoracic echocardiography revealed an aortic valve opening area of approximately 0.9 cm², a peak systolic flow rate of 4.5 m/s, a differential pressure gradient of 82 mmHg, a left ventricular ejection fraction of 63%, a proximal aortic diameter of approximately 4.3 cm, and normal morphology and activity of the mitral and tricuspid valves. Aortic computed tomography angiography (CTA) results show thickening of the aortic valve with minimal calcification and dilation of the ascending aorta, with a diameter of 4.4 cm. Additionally, mild stenosis was observed in the anterior descending branch of the right coronary artery at the opening on coronary arteriography. Following a clear surgical indication, we considered performing mechanical aortic valve replacement alongside ascending aortic replacement or ascending aortoplasty for the patient. Ultimately, the patient chose ascending aortoplasty due to personal factors. The surgery was performed through a median sternotomy under moderate hypothermic cardiopulmonary bypass. After cardiac arrest, the aortic valve was excised through the aortic incision, and a mechanical valve prosthesis was placed, confirming that the valve leaflets opened and closed properly. A tapered resection of the dilated aortic wall was performed along the aortic incision, and the aortic incision was closed with a continuous suture using 5–0 Prolene thread. Postoperative examination revealed optimal functioning of the mechanical valve. Moreover, the ascending aorta exhibited satisfactory flow. The patient was discharged 16 days postsurgery. During the six-year follow-up period, the patient attended regular outpatient reviews at our center, where laboratory tests and transthoracic echocardiography results showed no significant abnormalities. Coagulation function was maintained within the acceptable range required after mechanical valve replacement.

**Figure 1:**
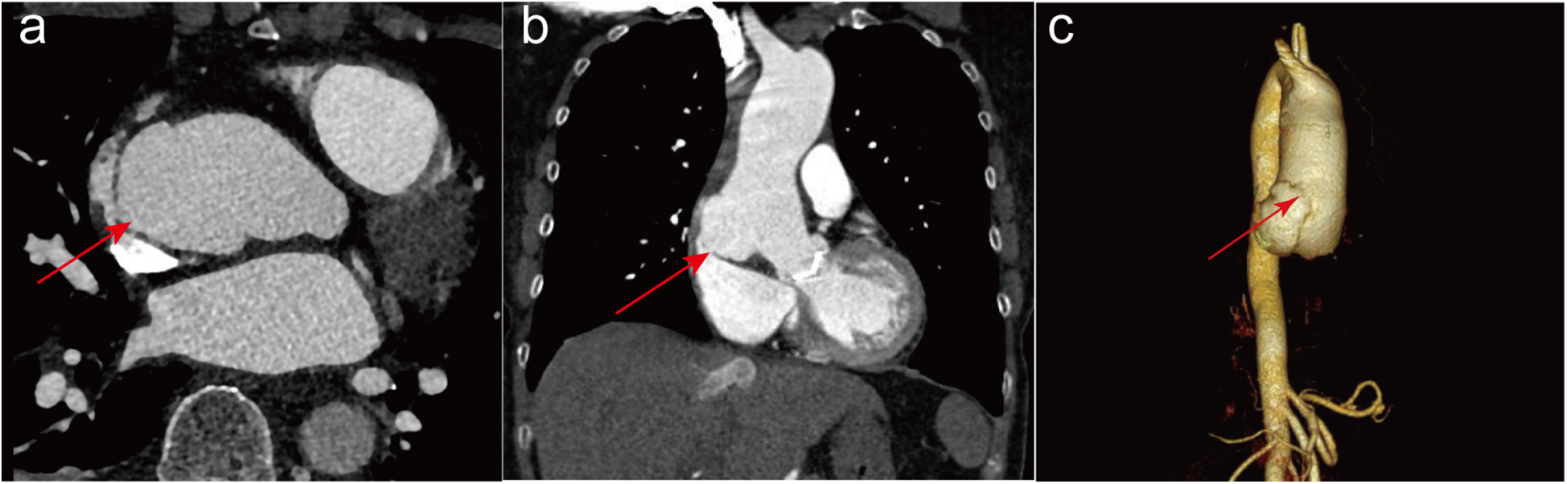
The imaging tests conducted on patients after admission to the hospital. Preoperative computed tomography angiography of the thoracic and abdominal aorta in both transverse **(a)** and longitudinal **(b)** sections showed a significant rupture at the ascending aorta, with the rupture connecting to the right atrium as indicated by the red arrow. Three-dimensional CTA with red arrows also shows a significant rupture in the ascending aorta **(c)**.

Six years after the operation, the patient sought admission to the emergency department of our hospital and presented with intermittent chest tightness and pain, coupled with weakness and dizziness. A transthoracic echocardiogram revealed a rupture on the right posterior wall of the ascending aorta extending into the right atrium. The ascending aorta exhibited aneurysmal dilation, with a proximal diameter of approximately 5.1 cm, indicating slight expansion compared to six years ago. The mechanical aortic valve exhibited normal function, characterized by systolic blood flow without significant acceleration, a peak flow rate of 3.3 m/s, and a differential pressure gradient of 44 mmHg. The left ventricular ejection fraction was measured at 65%. Additionally, the patient presented with a combination of moderately severe tricuspid insufficiency, moderate pulmonary hypertension, and a moderate to large amount of systolic regurgitant signal at the tricuspid orifice, featuring a peak flow rate of 3.1 m/s and a pressure gradient of 39 mmHg. Upon admission to the hospital, the patient underwent emergency room CTA of the thoracic and abdominal aorta. Imaging revealed limited bulging and protruding shadows measuring approximately 38*18 mm at the right edge of the lower section of the ascending aorta. The tip of this bulging structure was suspected to be connected to the right atrium, which appears to be flattened due to compression (Figures 1a,b), and a three-dimensional CTA likewise showed a significant rupture at the ascending aorta (Figure 1c). Moreover, the patient exhibited an international normalized ratio of 2.54 s, a prothrombin time of 24.9 s, and an elevated N-terminal pro-B-type natriuretic peptide level of 4030.0 pg/ml. Ultimately, owing to acute right heart failure resulting from the influx of blood from the ascending aorta into the right atrium, the patient was subsequently transferred to our cardiovascular surgery department for further treatment.

Following the confirmation of the absence of contraindications to surgery, we proceeded with ascending aortic replacement using a median sternotomy combined with a right inguinal incision. Subsequently, we initiated extracorporeal circulation by cannulation through the femoral artery, superior vena cava, and inferior vena cava. When circulatory arrest was achieved circulatory arrest through whole-body mild hypothermia, the surgical procedure commenced with removal of the pseudoaneurysm. Subsequently, the right atrial rupture was meticulously closed. The proximal portion of the ascending aorta was then trimmed, and the concluding phase involved the execution of ascending aortic replacement. Next, evaluation of tricuspid valve function was carried out through a right atrial incision, and the water injection test demonstrated the absence of discernible regurgitation. Following the closure of the right atrial incision, we disengaged from the extracorporeal circulation and meticulously closed the thoracic cavity using layer-by-layer sutures. Intraoperative transesophageal ultrasonography revealed unimpeded blood flow in the artificial vessel after ascending aortic replacement, patent coronary artery openings on both the right and left sides, and no discernible abnormalities subsequent to the repair of the right posterior wall breach of the ascending aorta. No discernible signal was detected during the diastolic phase of the aortic valve. Following the procedure, the patient was transferred to the cardiac care unit.

**Figure 2:**
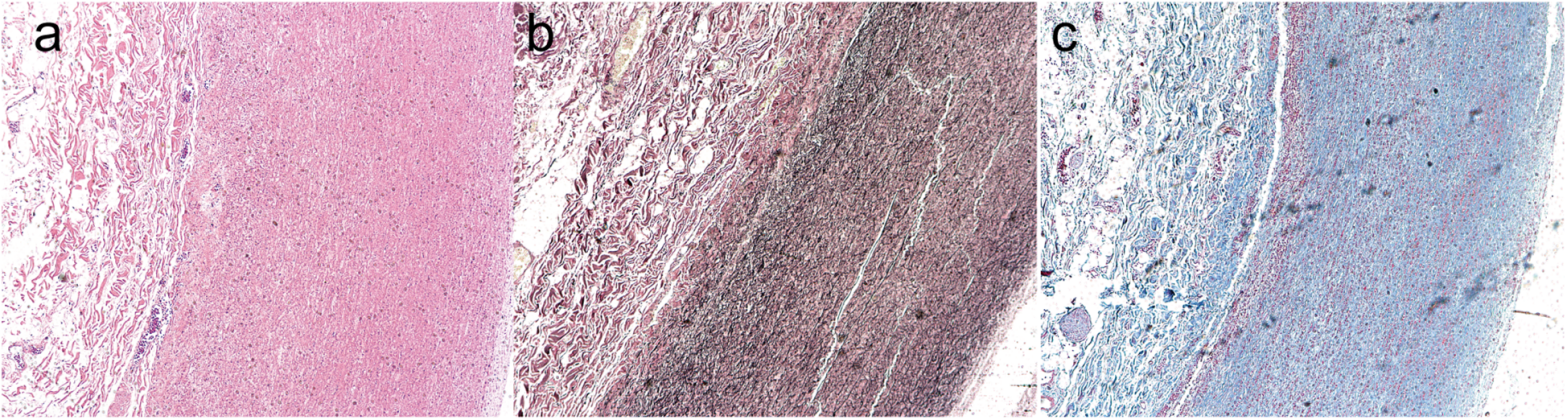
The postoperative patients’ aorta underwent pathological section staining. Hematoxylin-eosin staining **(a)**, Masson's trichrome staining **(b)**, and Verhoeff-van Gieson elastic staining **(c)** collectively revealed histological features indicative of smooth muscle cell disorganization, accompanied by a discernible decrease in elastin and collagen fiber density.

On the second postoperative day, a bedside transthoracic echocardiogram revealed the patent status of the artificial ascending aortic vessel, with a peak systolic flow at the aortic valve orifice measuring 2.6 m/s and a differential pressure gradient of 27 mmHg. Moreover, there was a normalization of tricuspid valve morphology and activity. After the surgery, sections of the patients' aorta were subjected to pathological staining for analysis. Hematoxylin-eosin staining (Figure 2a), Masson's trichrome staining (Figure 2b), and Verhoeff-van Gieson elastic staining (Figure 2c) collectively revealed histological features indicative of smooth muscle cell disorganization, accompanied by a discernible decrease in elastin and collagen fiber density. Prior to discharge, the patient demonstrated satisfactory cardiac function without any cardiovascular complications. Subsequent regular reviews at our outpatient clinic revealed no significant abnormalities in the patient's condition.

## Discussion

BAV is a highly heritable autosomal dominant genetic disorder, with disease progression, patients with BAV may experience aortic stenosis, aortic complications, infective endocarditis, and congestive heart failure (4). Long-term follow-up studies indicate that the probability of aortic dilation in BAV patients can be as high as nearly 50% (5). Furthermore, the risk of aortic aneurysm in BAV patients is significantly higher than that in the general population (2). Studies confirm that even without accompanying aortic valve stenosis or regurgitation, ascending aortic dilatation can be an early complication in BAV patients, which explains why ascending aortic dilatation can occur as early as the fetal period in these patients (6). A considerable percentage of individuals necessitating aortic valve replacement concurrently exhibit dilatation of the ascending aorta. If left unaddressed, this condition may give rise to potentially fatal aortic adverse events, as indicated by pertinent research findings (7). Winkler et al. paired 120 patients who underwent simple aortic valve replacement with 40 patients who underwent aortic valve replacement along with ascending aortic replacement, employing a propensity score matching ratio of 3:1. Their conclusive findings assert that the amalgamation of ascending aortic replacement does not augment surgical morbidity or mortality rates (8). In a parallel fashion, Idrees et al. employed propensity scores to pair 647 sets of patients, revealing that the execution of prophylactic aortic replacement concurrently with cardiac surgery involving mild concomitant aortic disease does not increase the risk of mortality (9). This outcome substantiates the safety associated with simultaneous aortic replacement during heart valve surgery. Furthermore, aortic lesions in patients with BAV exhibit abnormal hemodynamics and are predisposed to evolve into dilated aortic lesions in a more pronounced manner than those in TAV patients (10, 11). According to the 2022 American College of Cardiology/American Heart Association guidelines, surgical replacement of the ascending aorta is recommended for individuals with BAV and an aortic diameter equal to or exceeding 5.5 cm. Simultaneously, the guidelines posit that concurrent replacement of the ascending aorta is deemed a reasonable course of action for patients, necessitating valve replacement due to valve dysfunction, provided that the aortic diameter reaches or exceeds 4.5 cm (12). Nonetheless, empirical evidence has demonstrated that a substantial number of acute type A aortic dissections manifest at dimensions below the aforementioned threshold. Ascending aortic replacement may be considered in patients with BAV combined with ascending aortic dilatation, even if the aortic diameter is less than 5.0 cm (13). Moreover, magnetic resonance imaging studies have conclusively revealed a significantly greater prevalence of both ascending aortic diameter and ascending aortic dilatation in patients diagnosed with BAV than in those diagnosed with TAV (14). Investigations have indicated that individuals with BAV exhibit thinner elastic lamellae in the aorta along with a greater interlamellar distance when juxtaposed with patients harboring TAV (15). These structural distinctions may collectively contribute to accelerated progression of aortic pathology and increased vulnerability to adverse aortic events in individuals with BAV. The likelihood of adverse aortic events following isolated aortic valve replacement in individuals with mild to moderate ascending aortic dilatation is relatively low (16). However, due to the potential for aortic dissection following aortic dilation, prophylactic surgery remains necessary (17). In summary, even if the ascending aortic diameter in BAV patients has not reached the surgical threshold, surgeons may still reasonably consider ascending aortic replacement based on the individual patient's condition and actual clinical circumstances.

In accordance with the surgeon's clinical expertise and adherence to prevailing guideline criteria, none of the established surgical thresholds for ascending aortic replacement were met. Consequently, the chosen intervention involved aortic valve replacement concomitant with ascending aortoplasty. While ascending aortoplasty yields positive early-stage results, effectively slowing aortic diameter growth, and has the advantages of being safe and effective, with fewer complications and a lower mortality rate, patients who undergo BAV still have problems with re-expansion. Hence, a prolonged postoperative follow-up is necessary (18, 19).

Individuals with BAV and concurrent ascending aortic dilatation should undergo early surgical intervention. The selection of appropriate surgical modalities and personalized treatment plans is crucial for mitigating the risk of severe complications. This case also reminds us that more aggressive treatment of the dilated ascending aorta in practice may be a more sustainable and wise choice.

## Data Availability

The original contributions presented in the study are included in the article/Supplementary Material, further inquiries can be directed to the corresponding author.
